# Supplementary figures and images for: Motion magnification analysis of microscopy videos of biological cells
Source: PLoS One. 2020 Nov 5;15(11):e0240127. doi: 10.1371/journal.pone.0240127 (PMC7644077; doi:10.1371/journal.pone.0240127)

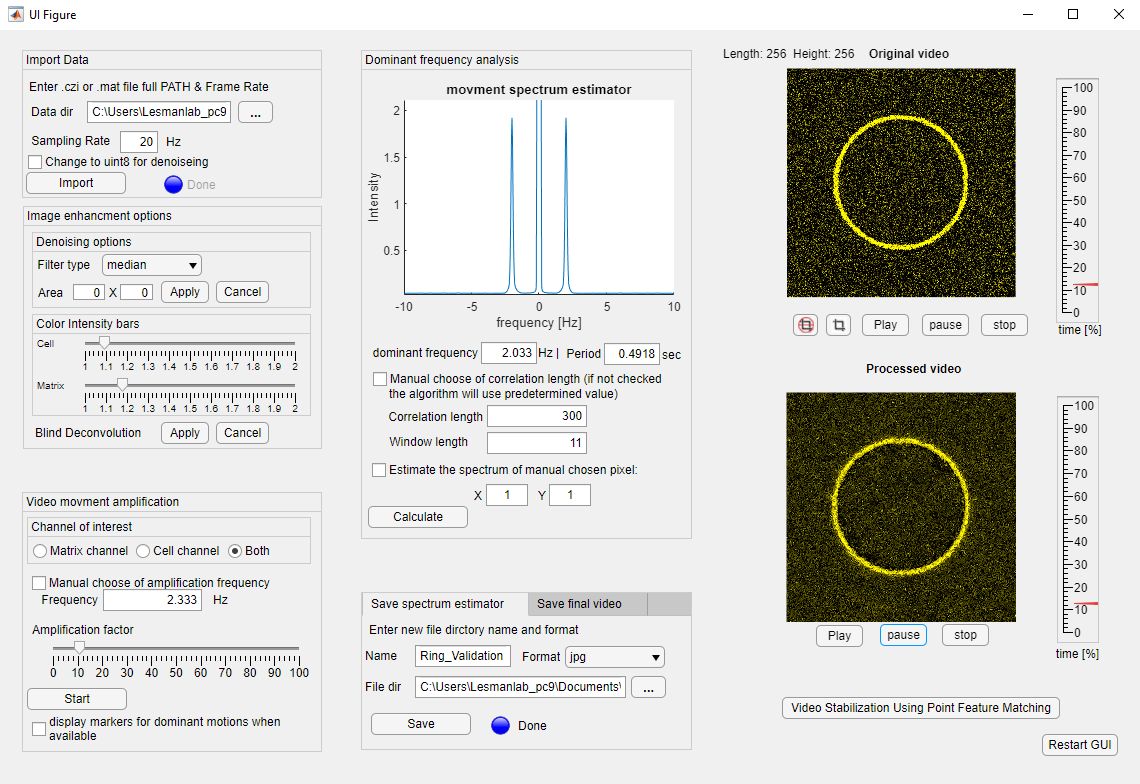


**S1 Figure –** Typical view of the GUI (Graphical User Interface).

Supplement: S1 Fig — (DOCX) [file pone.0240127.s005.docx]

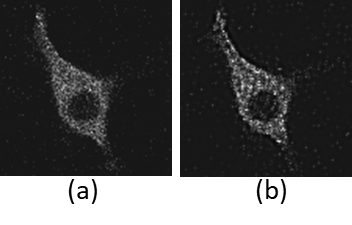


**S2 Figure –** Typical frame of example video before (a) and after (b) deconvolution process.

Supplement: S2 Fig — Typical frame of example video before (a) and after (b) deconvolution process. (DOCX) [file pone.0240127.s006.docx]
