# Supplementary material for: Motion magnification analysis of microscopy videos of biological cells
Source: PLoS One. 2020 Nov 5;15(11):e0240127. doi: 10.1371/journal.pone.0240127 (PMC7644077; doi:10.1371/journal.pone.0240127)
Supplement: S3 Fig — Acquired image in a confocal microscope fibroblast cell (colored green pixels) embedded in fibrin gel (colored red pixels) (a); Edge detection: cell contour pixels detected (b); Spectrum Estimation: video power spectrum analysis for the edge pixels (c). (DOCX) [file pone.0240127.s007.docx]

**
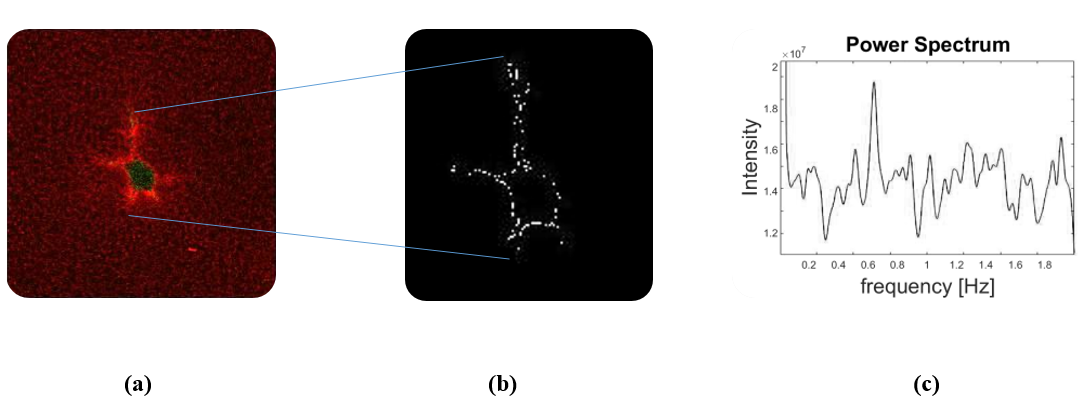
**

**S3 Figure –** Confocal microscope image of a fibroblast cell (colored green pixels) embedded in fibrin gel (colored red pixels) (a); Edge detection: cell contour pixels detected (b); Spectrum Estimation: video power spectrum analysis for the edge pixels (c).
