# Supplementary material for: Motion magnification analysis of microscopy videos of biological cells
Source: PLoS One. 2020 Nov 5;15(11):e0240127. doi: 10.1371/journal.pone.0240127 (PMC7644077; doi:10.1371/journal.pone.0240127)

**S4 Figure –** Phase of movement. Expansion designated by blue pixels, and contraction by red pixels, delineated on a kymograph of a short segment of a ring changing its size periodically.
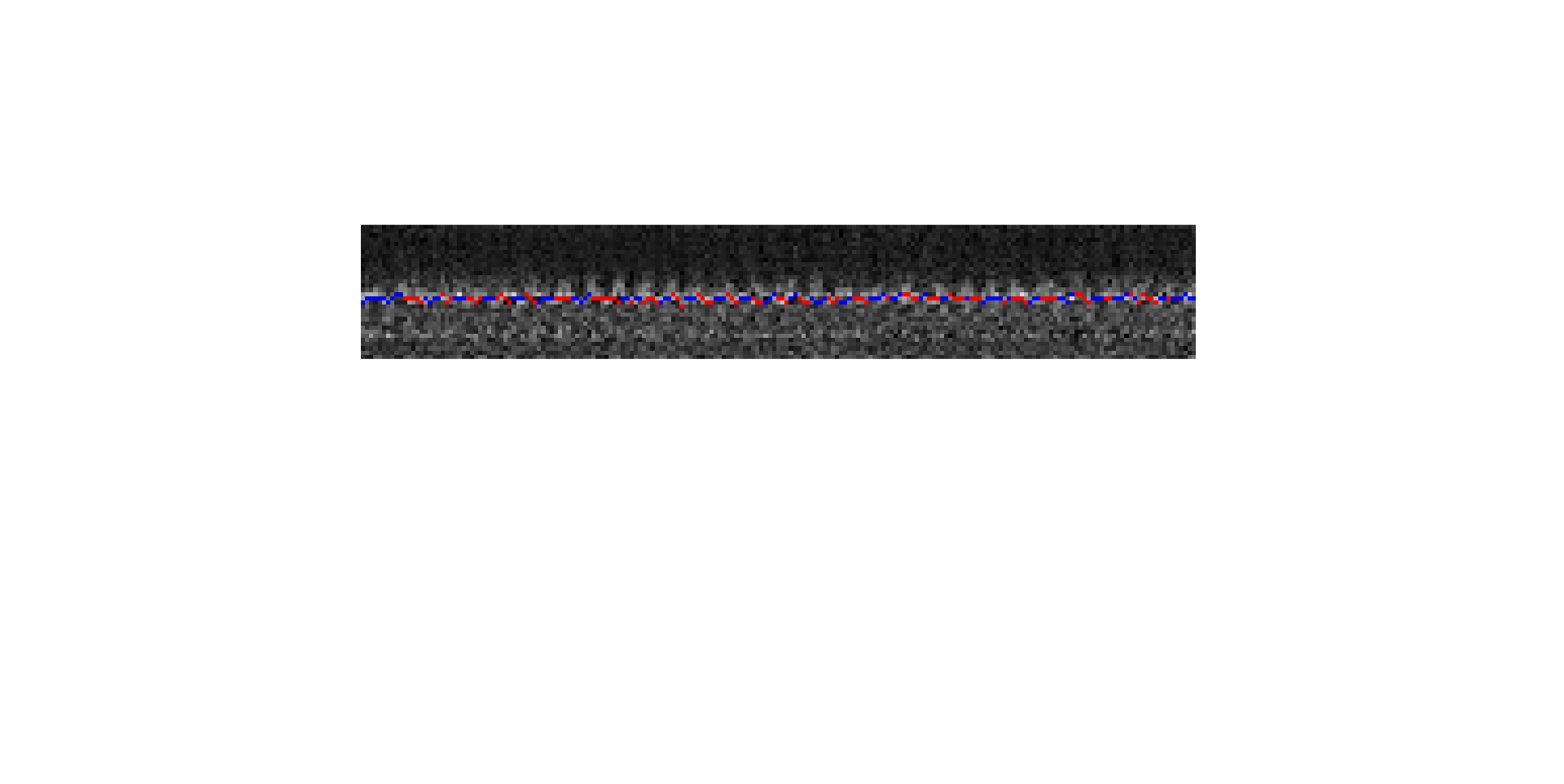

Supplement: S4 Fig — Expansion designated by blue pixels, and contraction by red pixels, delineated on a kymograph of a short segment of a ring changing its size periodically. (DOCX) [file pone.0240127.s008.docx]
