# Supplementary material for: Motion magnification analysis of microscopy videos of biological cells
Source: PLoS One. 2020 Nov 5;15(11):e0240127. doi: 10.1371/journal.pone.0240127 (PMC7644077; doi:10.1371/journal.pone.0240127)
Supplement: S5 Fig — (A) Live cells with a dominant peak is at 0.76 Hz, and (B) dead cells (PFA fixated) without a distinguished dominant peak. (DOCX) [file pone.0240127.s009.docx]

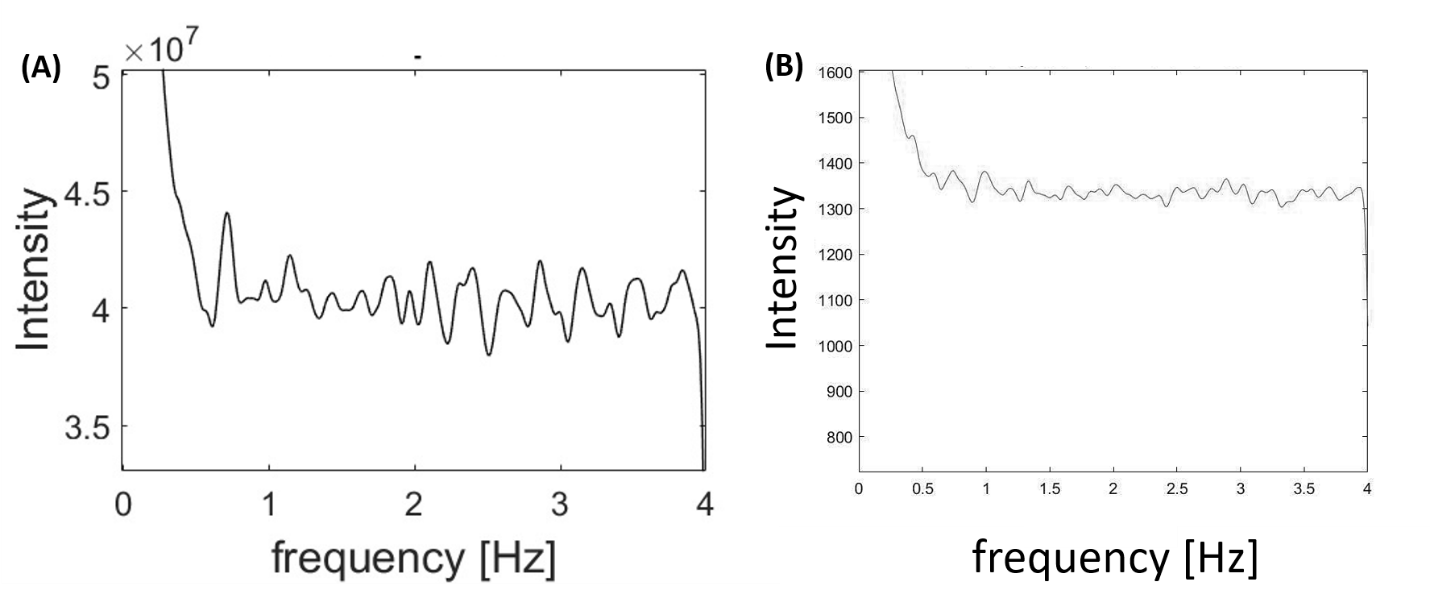


**S5 Figure** – Representative power spectrum of fibroblast cells cultured on top of a 2D glass dish. (A) Live cells with a dominant peak is at 0.76 Hz, and (B) dead cells (PFA fixated) without a distinguished dominant peak.
