# Supplementary material for: Motion magnification analysis of microscopy videos of biological cells
Source: PLoS One. 2020 Nov 5;15(11):e0240127. doi: 10.1371/journal.pone.0240127 (PMC7644077; doi:10.1371/journal.pone.0240127)
Supplement: S6 Fig — (A) Live cells with dominant peak at 1.28 Hz, and (B) Dead cells (PFA fixated) without a distinguished dominant peak. (DOCX) [file pone.0240127.s010.docx]

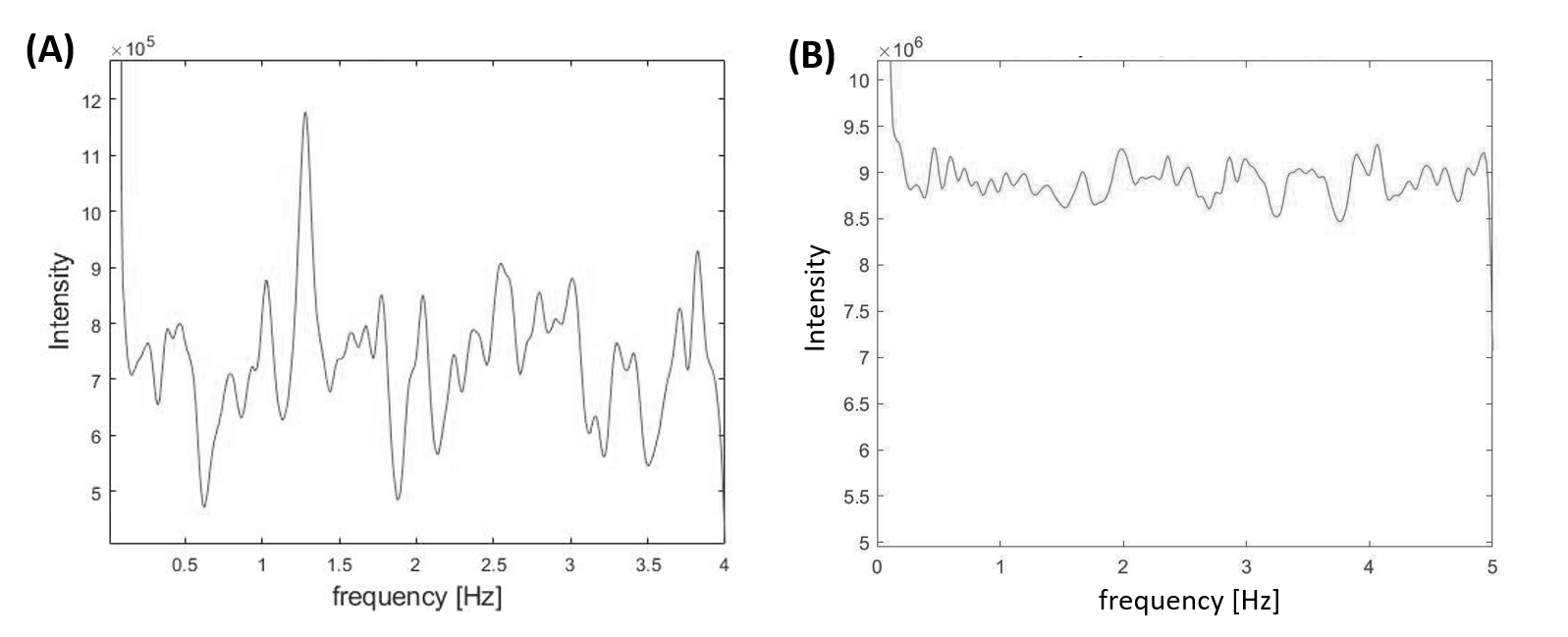


**S6 Figure** – Representative power spectrum of fibroblast cells embedded within a 3D fibrin gel. (A) Live cells with dominant peak at 1.28 Hz, and (B) Dead cells (PFA fixated) without a distinguished dominant peak.
