# Supplementary material for: Motion magnification analysis of microscopy videos of biological cells
Source: PLoS One. 2020 Nov 5;15(11):e0240127. doi: 10.1371/journal.pone.0240127 (PMC7644077; doi:10.1371/journal.pone.0240127)
Supplement: S7 Fig — The reference image for spectrum calculation is the whole image (182 × 286 pixels). (DOCX) [file pone.0240127.s011.docx]

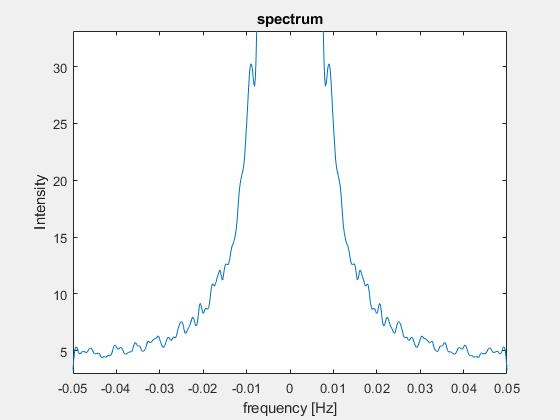


0.009 Hz

**S7 Figure**  – Power spectrum of live MDA-MB-231 cells on top of micropillars. The reference image for spectrum calculation is the whole image (182 × 286 pixels).
