# Supplementary material for: Motion magnification analysis of microscopy videos of biological cells
Source: PLoS One. 2020 Nov 5;15(11):e0240127. doi: 10.1371/journal.pone.0240127 (PMC7644077; doi:10.1371/journal.pone.0240127)
Supplement: S8 Fig — The figure compares the power spectra of three windows (70 × 90 pixels wide) that focus on micropillars: (i) 1st window, micropillars under a live cell (yellow); (ii) 2nd window, far-field micropillars without cells (red), taken from the same video of the live cells (yellow). (iii) 3rd window, micropillars under a fixated cell (blue), taken from the ‘dead cells’ video. Note that the blue and red spectra are very similar, especially in the high frequencies and both are very different (weaker) than the yellow spectrum of micropillars under live cells. Here, the reference image for spectrum analysis is smaller (70 × 90 pixels) than the one shown on S7 Fig, in order to compare live MDA-MB-231 cells and far-field micropillars without any cells, in the same video. (DOCX) [file pone.0240127.s012.docx]

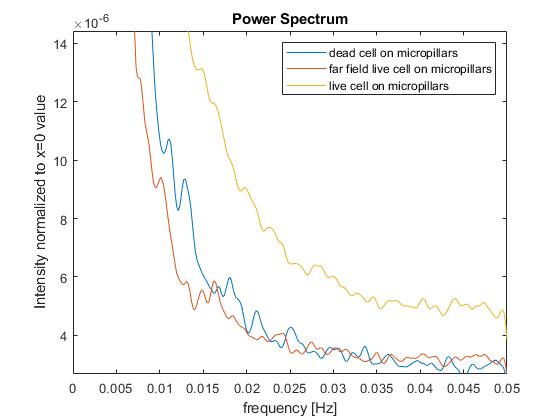


**S8 Figure**  – Power spectra of cells on micropillars. The figure compares the power spectra of three windows (70 × 90 pixels wide) that focus on micropillars: (i) 1^st^ window, micropillars under a live cell (yellow); (ii) 2^nd^ window, far-field micropillars without cells (red), taken from the same video of the live cells (yellow). (iii) 3^rd^ window, micropillars under a fixated cell (blue), taken from the ‘dead cells’ video. Note that the blue and red spectra are very similar, especially in the high frequencies and both are very different (weaker) than the yellow spectrum of micropillars under live cells. Here, the reference image for spectrum analysis is smaller (70 × 90 pixels) than the one shown on Fig. S7, in order to compare live MDA-MB-231 cells and far-field micropillars without any cells, in the same video.
