# Supplementary material for: Motion magnification analysis of microscopy videos of biological cells
Source: PLoS One. 2020 Nov 5;15(11):e0240127. doi: 10.1371/journal.pone.0240127 (PMC7644077; doi:10.1371/journal.pone.0240127)
Supplement: Attachment — Submitted filename: revision_letter_AL.pdf [file pone.0240127.s013.pdf]

School of Mechanical Engineering

בית הספר להנדסה מכאנית

Iby and Aladar Fleischman
Faculty of Engineering
Tel Aviv University

הפקולטה להנדסה
ע"ש איבי ואלדר פליישמן
אוניברסיטת תל-אביב

Re: Manuscript ID PONE-D-20-13055

Journal: PLOS ONE

Title: " Motion Magnification Analysis of Microscopy Videos of Biological Cells "

Author(s): O. Shabi, S. Natan, A. Mukherjee, O. Tchaicheeyan, H. Wolfenson, N. Kiryati, A. Lesman

Dr. Jorge Bernardino de la Serna

Date: September 6th, 2020

Academic Editor

Plos One

Dear Dr. Bernardino de la Serna,

We would like to thank you for handling the submission of our manuscript and sending us the referee reports on our manuscript "**Motion Magnification Analysis of Microscopy Videos of Biological Cells**". We thank the reviewers for taking the time to provide us with their helpful suggestions, which have led to improvement in the manuscript. Specifically, based on the referee's suggestions, we conducted another control experiment ('Dead' vs. 'Alive' cells), included three new Supplementary Figures, and have modified several parts of the manuscript.

All changes to the manuscript are highlighted in a yellow marker. Below, we copy (in black) the reports written by the reviewers, and follow them by our detailed point-to-point response (in blue) to all of the issues raised by the reviewers.

We hope that with this revision and explanations our updated manuscript can be accepted for publication in *Plos One*. If any further changes will be required, please do not hesitate to contact us.

We thank you in advance for your consideration.

Sincerely yours,

Ayelet Lesman and co-authors

Reviewer #1:

The manuscript titled "Motion Magnification Analysis of Microscopy Videos of Biological Cells" has reported a video processing method to detect micromovement of cells. The authors demonstrated the utility of the method by analyzing periodic movements of fibroblast cells in 2D and 3D culture environments, and micropillar interactions with MDA-MB-231 cells. Their conclusions are clear and are appropriately supported by the data. Therefore, I recommended the manuscript for publication after minor revisions shown as below.

1. In figure 1, the steps description(I-IV) in the figure legend is not consistent with that in the figure. Please do the alignment.

Answer:

We have modified Fig. 1 legend as follows:

Figure 1 – Overview of the method, including the steps: (I) Obtain an Input Video (time-lapse sequence) from a confocal microscope, and choose a plane from the z-stack to analyze. A representative 'y-t plot' of pixel intensity versus time (aka, kymograph plot) is also shown below for the intersecting arrow line in the image above. The example shows confocal image of an isolated fibroblast cell (dark green pixels) embedded within Fibrin hydrogel 3D matrix (red); next, (II) Preprocessing of the input video for denoising, reversing the distortion of the microscope and video stabilization, a similar 'y-t plot' is also shown below for the same line as in the first step; next, (III) Finding dominant movement frequencies using spectrum estimation. The power spectrum in the example exhibits a detected dominant frequency at 1.28 Hz; next, (IV) motion magnification at a chosen dominant frequency (e.g., 1.28 Hz) using EVM, the kymograph below corresponds (as in 1st & 2nd steps) to pixel intensity change over time and now a wavy pattern is noticeable for some pixels; next (V) Detecting motion directors (in-plane) and motion phase (red/blue for a given director).

Instead of the original:

Figure 1 – Overview of the method, including the steps: (I) deconvolution with the confocal microscope PSF, (II) finding dominant movement frequencies using spectrum estimation, (III) motion magnification using EVM, and (IV) motion detection and finding the motion directors. The example shows confocal images of an isolated fibroblast cell (green) embedded within Fibrin 3D matrix (red), with a detected dominant frequency at 1.28Hz.

2. Experimental details about cell culture in fibrin gel, culture dishes and micropillars need to be provided in method.

Answer:

We have added experimental details to the method section that now reads:

"**2.1.1 Cell Culture**

Actin-GFP 3T3 fibroblast cells (a gift from Prof. Scott E. Fraser, USC, Los Angeles, CA) were cultured in DMEM supplemented with 10% fetal bovine serum, nonessential amino acids, sodium pyruvate, l-glutamine, 100 units/ml penicillin, 100 µg/ml streptomycin, and 100 µg/ml neomycin (all the materials supplied by Biological Industries, Kibbutz Beit Haemek, Israel), in a 37°C humid incubator.

2.1.2 3D Fibrin Gel Preparation

Actin-GFP 3T3 fibroblast cells (5×10^3 cells) were mixed with 10 µl of a 20 U/ml Thrombin solution (Omrix Biopharmaceuticals). Then, 10 µl of a 10 mg/ml fluorescently labeled fibrinogen (Omrix Biopharmaceuticals) suspension - labeled with Alexa Fluor 546 as we described previously [49] - was placed on a #1.5 coverslip in a 35-mm dish (MatTek Life Sciences) and mixed with the cells+Thrombin suspension. The resulting fibrin gel was placed in the incubator for 20 min to polymerize, after which, warm medium was added to cover the gel.

2.1.3 2D Rigid Substrate Sample Preparation

Actin-GFP 3T3 fibroblast cells were sparsely distributed on glass bottom dishes, culture treated, uncoated (14 mm glass diameter, #1.5 Coverslip, MatTek, Ashland, MA). We used paraformaldehyde (PFA)-fixated cells as a control group (9 sample repetitions for each group). The cells adhered directly to the glass dishes.

2.1.4 2D Cells on top Flexible Micropillars

Live MDA-MB-231 epithelial cells (human breast adenocarcinoma source) were obtained from Prof. Yuval Shaked (Technion, Israel). These cells were cultured at 37 °C in a 5% CO_2 incubator in DMEM supplemented with 10% fetal bovine serum, 100 IU/ml penicillin–streptomycin, 2 µM L-glutamine and 2 µM HEPES. One day before spreading experiments, cells were sparsely plated on a culture dish to minimize cell–cell interactions before re-plating on the pillars. The following day, cells were trypsinized using TrypLE (Biological Industries), centrifuged with growth medium, and then resuspended and pre-incubated in in HBSS buffer supplemented with 20 mM Hepes for 30 min before the experiment. Cells were then plated on top of the micropillars substrate, as previously reported by Wolfenson et al. [19, 50].

2.1.5 Time-lapse microscopy

2.1.5.1 Images of 3T3 fibroblast cells

Fibroblast cells were imaged with a Zeiss 880 (Axio Observer) confocal laser scanning microscope, equipped with a ×40, NA = 1.1 water immersion lens (Zeiss) and a 30-mW argon laser (wavelengths 488 and 514 nm). Throughout imaging, cells were maintained in a 37°C 5% CO_2 incubation chamber. Confocal z-stacks were acquired at time intervals as mentioned for each video.

2.1.5.2 Images of MDA-MB-231 cells

Time-lapse imaging of cells spreading on the pillars was performed as described previously [Feld, L. et al. (2020) 'Cellular contractile forces are nonmechanosensitive', Science Advances, 6(17), p. eaaz6997. doi: 10.1126/sciadv.aaz6997.]. Imaging was performed using an inverted microscope (Leica DMIRE2) at 37°C using a ×63, NA = 1.4, oil immersion objective. Bright-field images were recorded every 10 s with a Retiga EXi Fast 1394 charge-coupled device camera (QImaging). The microscope and camera were controlled by Micro-Manager software [Edelstein, A. et al. (2010) 'Computer control of microscopes using microManager', Curr Protoc Mol Biol 92, pp. 14.20.1–14.20.17. doi: 10.1002/0471142727.mb1420s92.]. To minimize photodamage to the cells, a 600-nm long-pass filter was inserted into the illumination path. "

Instead of the original:

"2.1 Biological Sample Preparation

Approximately 5×10^3 NIH 3T3-GFP-actin cells were seeded in 20 μl of fibrin gel, labeled with Alexa Fluor 546 as we described previously [49]. The gel was scanned at several time points post-seeding, using a confocal laser-scanning microscope (Zeiss LSM 880 lens ×40, water immersion), to capture isolated cells in the sample.

We also analyzed cells sparsely distributed on plastic culture dishes, and used paraformaldehyde (PFA)-fixated cells as control group (9 sample repetitions for each group).

Finally, we analyzed live cells cultured on micropillars substrates, using a system that was previously reported by Wolfenson et al. [19, 50]."

3. Although the authors performed comparison of oscillatory behavior between live and dead fibroblasts, I cannot find the spectra data for dead cells.

<u>Answer</u>:

We now include a representative power spectra of dead cells in 2D dish (Figure S5-B) and 3D gel (Figure S6-B) in the Supplementary Info.

Are they compared in 2D or 3D culture environment?

<u>Answer</u>:

We have added to the text in section 3.3 information both about 2D and 3D cultures and their spectra characteristics:

"have found that for live cells the value of the dominant frequency peak is 8% - 12% above the average peak height (8.2% in 2D; 9.2% in 3D) and the height of the dominant peaks found in videos of dead cells are only about 5%-8% above the average intensity (6.8% in 2D; 7.4% in 3D)."

The author also claimed that the motion frequency in 2D dishes is four times lower than in 3D hydrogel. Is it because of cell movement or the hydrogel movement?

Answer:

In 2D culture the cells are attached to the glass bottom and the stiff substrate does not transfer information and does not allow mechanical interaction between the relatively far away cells. In fibrin gels, cells embedded in the gel exert force and this force may be carried along relatively long distances in the elastic gel, and therefore oscillations at the proximity of one cell may be influenced by oscillations exerted by neighboring cells. We have added in the Discussion section (line 552, section 4.2) :

"We would remark that in 2D cultures the cells are attached to the glass bottom and the stiff substrate does not allow mechanical interaction between the relatively far away cells. In fibrin gels, cells embedded in the soft gel exert force which may be carried along relatively long distances in the elastic gel [Natan, 2020; Goren, 2020], and therefore oscillations at the proximity of a cell may be caused by oscillations of the same cell and those exerted by neighboring cells. Hence, some oscillatory movement may be mediated by neighboring cells and not necessarily by the cell itself. Furthermore, the relatively large mechanical compliance of the gel may allow the fast oscillatory action of the actomyosin machinery while the stiff glass bottom may halt the actomyosin machinery at isometric exertion (according to the force-velocity relationship, FVR [Cheng, 2017])."

To clarify this, control experiments (fixed cells in 2D and 3D culture) should both be provided. In addition, fixed MDA-MB-231 cells in micropillar movements should also be tested as a control.

Answer:

We show fixed cells in 2D (new Figure S5-B) and 3D cultures (new Figure S6-B). We also added a control experiment of fixated MDA-MB-231 cells on micropillars and added a spectra analysis in a new Fig S8 that compares the power spectra of three areas: micropillars under a live cell, far field micropillars without any cells ('live cells' video), and micropillars under a fixated cell ('dead cells' video).

4. In figure S7, is it a spectrum for fibroblasts or MDA-MB-231 cells?

Answer:

It is indeed MDA-MB-231 cells, we have changed the legend to the following:

"Figure S7 – Power spectrum of live MDA-MB-231 cells on top of micropillars"

5. Movie S1 is not accessible

Answer:

We will try to upload the movie again as a supplementary file.

6. Movie S3 and S4 are not necessary, better to provide figures for clarification.

Answer:

Instead of movies S3 and S4, representative power spectra are provided now as images in the Supplementary Info Figures S5 and S6. Note that because we removed the original Movies S3 & S4 we have enumerated Movies S5&S6 as S3&S4.

Reviewer #2: The authors present a very compelling analysis quantifying the oscillations that certain cells evert on their environments. The analysis tool is excellent, well explained, and well-validated, and the results are compelling and interesting.

This is the best quantification of this phenomenon of which the reviewer is aware, and the reviewer believes this to be an outstanding contribution that can be published in its current form.

If the authors choose to revise their discussion, it might make sense to connect with discussions of these oscillations in some additional contexts.

One is the well-known oscillations of the cells in a range of developmental contexts, beginning very early in development. A few nice references follow:

Sokolow, A., Toyama, Y., Kiehart, D.P. and Edwards, G.S., 2012. Cell ingression and apical shape oscillations during dorsal closure in Drosophila. Biophysical journal, 102(5), pp.969-979.

Hutson, M.S., Brodland, G.W., Ma, X., Lynch, H.E., Jayasinghe, A.K. and Veldhuis, J., 2014. Measuring and modeling morphogenetic stress in developing embryos. In Mechanics of Biological Systems and Materials, Volume 4 (pp. 107-115). Springer, Cham.

Durney, C.H., Harris, T.J. and Feng, J.J., 2018. Dynamics of PAR proteins explain the oscillation and ratcheting mechanisms in dorsal closure. Biophysical journal, 115(11), pp.2230-2241.

Answer:

We thank the reviewer for his positive feedback and his suggestions. We have added the following paragraph and references in the manuscript (Section 1, beginning line 27)

"On the organism level, one may mention as an example, the early phase of dorsal closure that is an important morphogenetic process during the embryonic development of Drosophila (fruit fly). There, apical cell-shape oscillations were observed in amnioserosa epithelial cells [Sokolow, 2012]. This pulsed - actomyosin-based - constriction frequency band lies between 2.4 and 9.0 mHz (periods of 110–500 s), and center at ~4.2 mHz fluctuations (~4 min period). The mechanics of cell oscillation is driven by biochemical signaling through an intracellular negative feedback loop where the dynamics of the PAR proteins that interact with each other ultimately regulate the apicomedial actomyosin assembly and disassembly with a period of ~4 min [Durney, 2018]. Laser microsurgery techniques were used to investigate observed dynamic oscillations in amnioserosa cells [Hutson,2014]. These oscillations are mostly out of phase in neighboring cells. Both the contraction and expansion phases of this cycle are largely cell autonomous [Hutson,2014]. Interestingly, actomyosin-based oscillations also have been observed in other biological systems at low-Reynolds number. For example, shape oscillations were observed in purified preparations of actin and myosin that exhibit spontaneous oscillations [Fujita, 1998].

Another possible context is the early work from the group of Elliot Elson on using tissue constructs as platforms for basic research and drug discovery-- these oscillations are discussed as appearing in ensemble measurements on 3D tissue constructs.

Answer:

We have added the following paragraph and references in the manuscript (Section 4.2, ending paragraph)

"Currently, tissue constructs with cells embedded in three-dimensional matrices that mimic the natural tissue serve as platforms for basic research, regenerative medicine and drug discovery. In such tissue constructs, force oscillations were detected, for example, as ensemble of beating cells movements [Elson, 2016]. Thus, the EVM tool may be used for the study of mechanobiology in such tissue constructs. It may also be used for the study of biochemical oscillations as exhibited, for example, by oscillatory enzyme kinetics in cyclic reactions [Qian, 2002]."

The current study is an outstanding and long sought quantification of these oscillations at the cellular level, and it might be nice to add the long history of efforts to see them to emphasize the importance and impact of the work.

Answer:

Indeed, adding a paragraph that will emphasize the importance of this work by detailing the long history of efforts to see and quantify oscillations at the cellular level, will benefit our article. However, it will also make the manuscript longer and we feel that this issue deserves a Review in its own right. We mention the possible impact of this tool throughout the Introduction.

However, the article as it stands covers much important ground, and these two angles are merely suggestions should the authors choose to incorporate them.

Great paper!

Answer:

Thank-you !
